# Commentary: Heart Failure with Preserved Ejection Fraction Induces Beiging in Adipose Tissue

**DOI:** 10.3389/fphys.2016.00085

**Published:** 2016-03-07

**Authors:** Salvatore Chirumbolo

**Affiliations:** University Laboratories for Medical Research-Medicine D, Department of Medicine-Unit of Geriatry, University of VeronaVerona, Italy

**Keywords:** beige brite adipocytes, white adipose tissue, FGF21, heart failure, brown adipose tissue

Valero-Muñoz et al. showed that heart failure with preserved ejection fraction (HFpEF) induced beiging in adipose tissue. They reported that in HFpEF brown adipose tissue (BAT) reduced the expression of some browning markers, such as uncoupling protein 1 (ucp-1), cell death-inducing DFFA-like effector a (cidea), and epithelial V-like antigen (eva), which were yet expressed by white adipose tissue (WAT) during beiging (Valero-Muñoz et al., 2016).

Taking into account the complex role exerted by beige, brown and WAT in heart physiology, this article raised some comments about the role of these adipocytes in heart failure and circulation. The reduction of brown markers in HFpEF, apparently circumvented by WAT beiging, suggests that a new homeostatic mechanism probably occurred in the relationship between adipose tissue and heart, where a possible role of browning the adipocyte depots is exerted by natriuretic peptides and the activity of the sympathetic nervous system (Collins, 2014). The main concern is if these beige adipocytes are to be considered either friends or foes in this relationship (Hassan et al., 2012). One possible hypothesis, though initially speculative, is that adipocytes undergoing beiging or browning were considered a “positive” response to myocardial injury, particularly in heart failure and coronary artery disease (CAD; Chirumbolo, 2016). Adipose stromal cells and dissociated BAT have been shown to generate cardiomyocyte-like cells and this was reported even for mature adipocytes (Jumabay et al., 2010). The mechanism might be induced by nitrogen-driven species, which are released particularly during heart failure (Roberts et al., 2015). At least for laboratory animals, natriuretic peptides, while interacting with their G-protein coupled receptors and activating endothelial NOS by a calcium calmodulin-dependent mechanism, increase nitric oxide production and consequently the release of further natriuretic peptides, which in turn should induce adipocyte beiging (Neinast et al., 2015).

However, the paper by Valero-Muñoz et al. did not fully explain the role of this beiging mechanism, as the meaning of WAT undergoing a browning-like mechanism and generating beige/brite adipocytes in HFpEF yet appears puzzling. In *in vitro* 3T3-L1 cell line, chronic treatment with catecholamines seems to exacerbate the “beige”-like phenotype, in agreement with some evidence reporting that catecholamines induce a thermogenic response in brown adipocytes. This issue may suggest that both immune stressors and mitochondrial turn over are involved in the mechanism. Actually, during heart injury, adipocytes may serve also to modulate mitochondrial biogenesis through the PGC-1α/PPARs pathway and induce cell de-differentiation and/or re-programming to promote cardiomyocyte renewal. In this perspective, beige adipocytes may be considered as an intermediate phenotype able to regulate the homeostatic balance between BAT and WAT, and should be responsive to several players of injury, inflammation and stress, such as prostaglandins, particularly PGE_2_, which notoriously increases in heart failure (García-Alonso and Clària, 2014). The role of adipose tissue in heart function might not be restricted to the adipocyte contribution to the metabolic and energetic balance, therefore. The model, summarized in Figure 1, would suggest that adipocytes are continuously transformed and recruited to regulate energetic dustress (particularly at the mitochondria/calcium signaling level), inflammation and stress and further provide tissue renewal. Lipidic impairment and stress may lead this balance to WAT.

**Figure 1:**
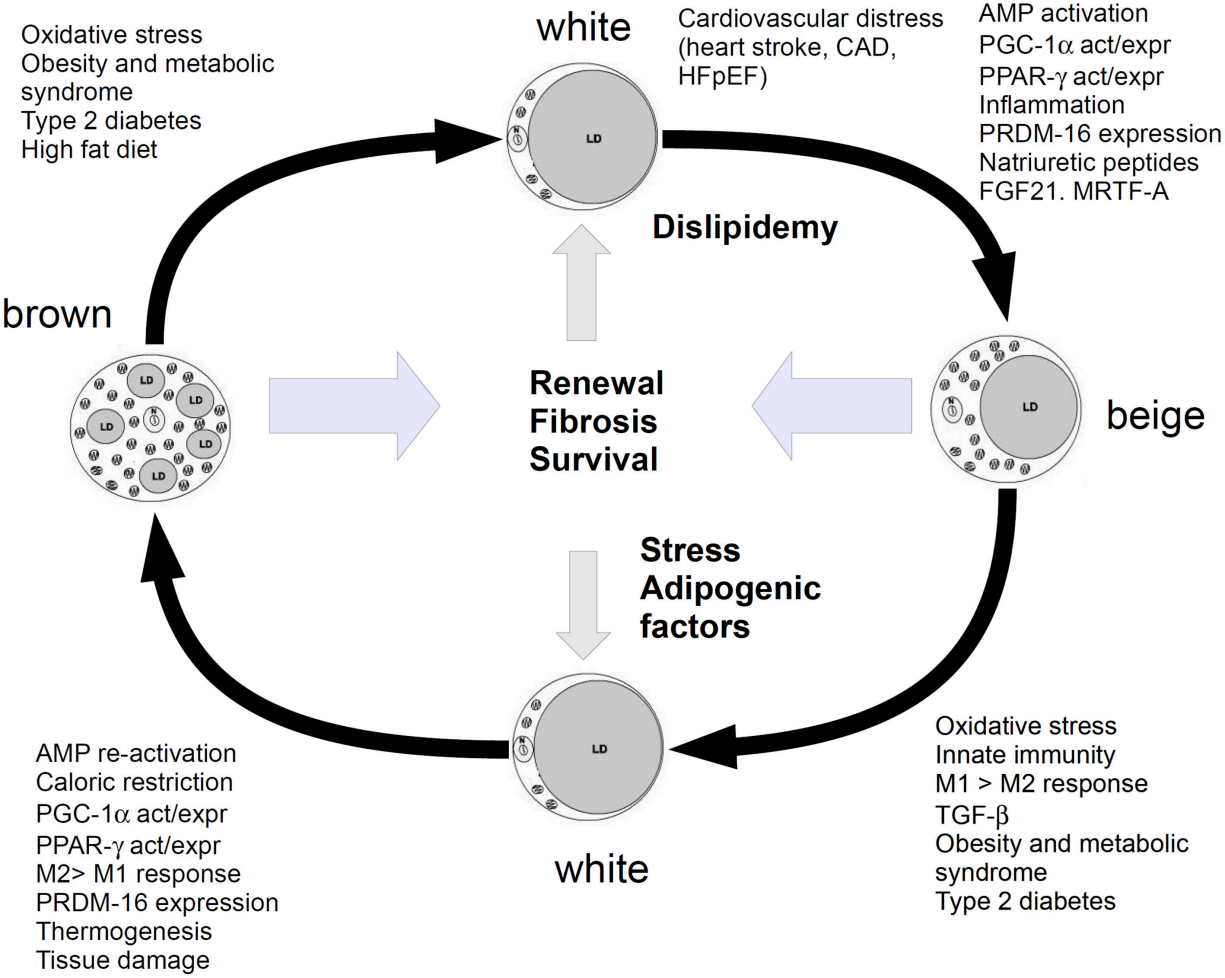
**Cartoon showing the relationship between white, beige (brite) and brown adipocytes**. Metabolism, energetic and inflammatory-stress response factors, shift white adipose to beiging and further stimulate it to browning. WAT is promoted by dysfunction in glucose/lipid homeostasis, which leads to the chronic impairment of heart function. Beiging is a pro-repairing mechanism, induced by molecules and functions, which may lead to browning, if the organism is able to respond with a fibrotic/renewal (repair) mechanism, without entering a chronic inflammatory stage. This mechanism should serve to recover survival and anti-inflammatory functions and tissue renewal. Stress or high fat diet or dyslipidaemia and metabolic syndrome may shift this balance to WAT.

Furthermore, an interesting issue coming from the paper by Valero-Muñoz et al. reported that changes induced by HFpEF were associated with smaller sized adipocytes. Small adipocytes were described during inflammation also by our group (Zoico et al., 2014; Chirumbolo et al., 2015) and therefore related to inflammation signaling and innate immunity. During heart failure, the JAK-STAT signaling system regulates adipocyte response to inflammation by adjusting cell metabolism (Richard and Stephens, 2014; Lim and Lam, 2016). The inhibition of JAK signaling modifies white adipocytes into brown (Moisan et al., 2015). The possible repression of IFN-γ signaling and activation of Hedgehog signaling in JAK-inactivated adipocytes should contribute to the metabolic conversion white-brown with elevated expression of brown adipocyte markers. In this concern, beige or brown adipocytes may be fundamentally related to inflammatory-mediated signals, rather than the metabolic machinery, switching the conversion of some white adipocytes to beige ones (Moisan et al., 2015). Actually, the role of BAT has been recently related to the adipocyte response to inflammation aiming at preventing metabolic syndrome and cardiovascular disorders (Gaggini et al., 2015). According to this speculation, beiging should be related to signals coming from natriuretic peptides and mediators of inflammation, particularly in HFpEF (Valero-Muñoz et al., 2016). Plasma pro-brain natriuretic peptide (pro-BNP) has been associated with heart failure (Choi et al., 2012), together with adiponectin, which is released during inflammation (Lindberg et al., 2014). This perspective may suggest that inflammation and oxidative stress are fundamental triggers of adipose tissue beiging in the heart, besides to metabolic stress and AMPK activation.

To better focus onto these issues, it would be interesting if the authors should evaluate further markers, such as the white/beige co-regulator receptor interacting protein 140 (RIP140) and further deepen the role of immune cells, particularly M1 and M2 macrophages (Liu et al., 2015). The reduction of RIP140 in macrophages drives to adipocyte browning concomitant with an anti-inflammatory response (Liu et al., 2014). Inflammation drives WAT to beiging but this process may be much more complex than expected. Further molecules participate in this process, such as FGF21 or myocardin-related factor A (MRTF; Lee et al., 2014). Myocardin-related factor A (MRTF) has been reported to be involved in the origin of brite (beige) adipocytes from WAT (McDonald et al., 2015) but it is also involved in epicardium-derived progenitor cell (EPOC)-mediated origin of sub-endocardial pericytes (Trembley et al., 2015), from which new adipocytes may derive (Tran et al., 2012). A complex machinery of beiging/browning mechanisms, involving even microRNAs as regulators, drives the inflammatory and damage response to tissue repair, either fibrosis or renewal (Nakamura et al., 2013). While the paper by Valero-Muñoz et al. did not deepen the role of this beiging phenomenon in HFpEF, some suggestion arises about the possible meaning of these adipocytes in myocardial tissue repair.

A complex machinery of interactions between bone marrow precursors, pre-adipocytes, mature adipocytes and de-differentiated/mesenchymal progenitors, strongly suggests that the “adipose” compartment is a complex dynamic system that acts on several physiological districts to regulate energy balance, survival and tissue renewal (Hausman and Hausman, 2006). Brite/beige adipocytes, interacting with myocardial tissue, may have a tissue-renewal role, as adipose tissue derived stem cells, when conjugated with angiogenic factors, might exert a paracrine actions that may have therapeutic effect even in myocardial dysfunction (Madonna et al., 2015).

Further markers and investigations are needed to elucidate the role of adipose tissue and its beiging in heart function and heart failure.

## Author contributions

The author confirms being the sole contributor of this work and approved it for publication.

### Conflict of interest statement

The author declares that the research was conducted in the absence of any commercial or financial relationships that could be construed as a potential conflict of interest.
